# Supplementary material for: Chromothripsis during telomere crisis is independent of NHEJ, and consistent with a replicative origin
Source: Genome Res. 2019 May;29(5):737–49. doi: 10.1101/gr.240705.118 (PMC6499312; doi:10.1101/gr.240705.118)
Supplement: Supplemental Material [file supp_gr.240705.118_Supplemental_file_1.zip › contigs/annotated_contigs/DB80/contig.2.DB80_length_517_mean_cov_8.99419729207.docx]

**DB80_length_517_mean_cov_8.99419729207**

```
AGATTTCAGAAGCACTCAGCAGGACTGAGGATTGTTGATGAATTTCAAGGTTTTGTTGAATACTTTAAATTGCAACTAATTATAAGTCA
 >chr7:112837907-112838167 - E=7e-145 p=7e-03
TTGTTTAGAAATTGTTATACAACACCATTTTTGTAATTGATTTGTATAAACAGATATCGCATTGCATTATGTTTGGTTTTTGTGGTCTG

TAAATTCTTTAATTGCCACTACATTTTTCAAAGATCTCACCTATGACATTAACATCAACTTACCCATACTATGGTTTATAGT|AT|AGA
                                                                                     >c
CCTATCAATCTTTAAAGTATCTAAAGAAATTTACATTTTTCATACTTTCATCTTTTTTCATAAAAAGTATCTCAAACAAGATTAGATAT
hr7:112837064-112837319 - E=7e-142
AAAGCACAGATAATCCAAAGATAGCCTTGCTATTCTCTTGCTGGGTACAGACAATGATGCCAAGAAATATAGAAGAAATAGAATCTTCC

TGTTGATATATCAACCCAGTTTCAAGTGCATTTTTAAAAAGGAATCACTGCAGAGAGAAATAAATCAATGCTTT
```
